# Armed conflicts have an impact on the spread of tuberculosis: the case of the Somali Regional State of Ethiopia

**DOI:** 10.1186/1752-1505-4-1

**Published:** 2010-01-28

**Authors:** Abdi A Gele, Gunnar A Bjune

**Affiliations:** 1 Department of Social Science, Oslo University College, Oslo, Norway; 2 Section for International Health, Department of General Practice and Community Medicine, University of Oslo, Oslo, Norway

## Abstract

A pessimistic view of the impact of armed conflicts on the control of infectious diseases has generated great interest in the role of conflicts on the global TB epidemic. Nowhere in the world is such interest more palpable than in the Horn of Africa Region, comprising Ethiopia, Somalia, Eritrea, Djibouti, Kenya and Sudan. An expanding literature has demonstrated that armed conflicts stall disease control programs through distraction of health system, interruption of patients' ability to seek health care, and the diversion of economic resources to military ends rather than health needs. Nonetheless, until very recently, no research has been done to address the impact of armed conflict on TB epidemics in the Somali Regional State (SRS) of Ethiopia.

**Methods:**

This study is based on the cross-sectional data collected in 2007, utilizing structured questionnaires filled-out by a sample of 226 TB patients in the SRS of Ethiopia. Data was obtained on the delay patients experienced in receiving a diagnosis of TB, on the biomedical knowledge of TB that patients had, and the level of self-treatment by patients. The outcome variables in this study are the delay in the diagnosis of TB experienced by patients, and extent of self-treatment utilized by patients. Our main explanatory variable was place of residence, which was dichotomized as being in 'conflict zones' and in 'non-conflict zones'. Demographic data was collected for statistical control. Chi-square and Mann-Whitney tests were used on calculations of group differences. Logistic regression analysis was used to determine the association between outcome and predictor variables.

**Results:**

Two hundred and twenty six TB patients were interviewed. The median delay in the diagnosis of TB was 120 days and 60 days for patients from conflict zones and from non-conflict zones, respectively. Moreover, 74% of the patients residing in conflict zones undertook self-treatment prior to their diagnosis. The corresponding proportion from non-conflict zones was 45%. Fully adjusted logistic regression analysis shows that patients from conflict zones had significantly greater odds of delay (OR = 3.06; 95% CI: 1.47-6.36) and higher self treatment utilization (OR = 3.34; 95% CI: 1.56-7.12) compared to those from non-conflict zones.

**Conclusion:**

Patients from conflict zones have a longer delay in receiving a diagnosis of TB and have higher levels of self treatment utilization. This suggests that access to TB care should be improved by the expansion of user friendly directly observed therapy short-course (DOTS) in the conflict zones of the region.

## Background

Tuberculosis is a major public health problem in the world and the problem is particularly widespread in sub-Saharan Africa [1]. More than 80% of the people suffering from TB live in sub-Saharan Africa or in Asia [2], where spending on health care is low and access to drugs is limited. Although good TB programs in parts of Africa had an appreciable impact in the reduction of TB cases, military conflicts and civil strife in some countries play a major role in stalling TB control programs [3]. Many people die each year of TB in those parts of the world because various forms of war and low spending on health care deprive them of access to treatment [4]. Against this backdrop of neglect, it is little wonder that TB has been allowed to spread. Nonetheless, there is a lack of information on the impact of longstanding armed conflict in the SRS on TB epidemics in Ethiopia.

More than 191 million people lost their lives in the 20^th ^century due to armed conflicts [5]. Tuberculosis is known to be a major cause of morbidity and mortality in conflict settings [6-9]. When one combatant dies in the conflict, an additional 14 to 15 civilians die, mostly from preventable infectious diseases such as TB [10]. Conditions of war were associated with the rapid increase of morbidity and mortality from TB [7]. A review of the literature reported an increase in the incidence of TB during war years and excessive morbidity and mortality many years after the war [7]. For example, the TB mortality rate in Holland rose from 154 per 100,000 in 1915, to 180 per 100,000 in 1916, while the TB mortality rate increased by 50% in Berlin from 1916 to 1917 [6]. A prior study conducted in Nepal reported poor utilization of TB treatment and diagnostic services among war affected populations. This was mainly due to massive military campaigns, frequent curfews, and closures of services in conflict areas, and subsequently, to an increase in the prevalence of TB in the population [9].

Ethiopia ranks 7^th ^of the 22 countries with highest TB burden in the world [11]. The Somali Regional State of Ethiopia is an area suffering from a long running conflict. The conflict has severely undermined the ability of the public sector to deliver basic social services to most of its population. As a result, people in the region are not only exceedingly poor [12], but also bear a disproportionately high incidence of TB. In the year 2000, the incidence of pulmonary positive TB in the Somali Regional State of Ethiopia was noted at 175-250/100,000, which is much higher than the national level of 165/100,000 [13].

The population in the region overwhelmingly consists of Somali pastoral nomads; a migratory people whose livelihood is primarily based upon rearing livestock. These people migrate seasonally or episodically in search of grazing lands and water. Despite significant TB-related morbidity and mortality amongst the nomads of this region, the disease has been largely neglected [14]. We conducted a broad-based study that addressed socio-cultural attributes in the management and control of TB among Somali nomads in the SRS of Ethiopia from July to September 2007. As a part of this study, we documented the length of delay in receiving a diagnosis of TB that patients reported [14] and the barriers to TB care that they perceived (under publication). In this paper, we intend to examine the role of conflict in a regional TB epidemic by comparing patients from conflict zones in the region to patients from non-conflict zones with regard to the delay they experienced in the diagnosis of TB and the extent to which these patients utilized self treatments before the diagnosis of TB was made.

### Study area

The SRS is the second largest among the nine regions of Ethiopia, with a land area of 375,000 km^2 ^and an estimated population of 4 million people. Three different systems of livelihood exist in the SRS: these are; pastoralism, agro-pastoralism and urban [12]. An estimated 85% of regional populations earn their livelihoods from pastoralism or agro-pastoralism.

The Regional TB control program adopted the DOTS strategy, which is implemented through DOT clinics that are located in major towns. The private sector is very rare in the Somali Regional State of Ethiopia. Neither the private sector nor traditional healers are involved in the regional TB control program.

The region is characterized by longstanding conflict between government forces and local armed rebel forces, i.e., the Ogaden National Liberation Front (ONLF). Although the conflict had been simmering for years, new momentum occurred in early 2007 [15]. The SRS consists of 9 zones. However, most of the battles and war activities are concentrated in 5 zones, i.e., Dhagaxbur, Fiiq, Korahe, Gode and Wardheer. All of these 5 zones are overwhelmingly populated by pastoral nomads [15,16]. As a result, the nomadic populations are faced with restrictions of movement that prevent them from fully utilizing their traditional survival mechanisms and their access to health care [17]. Medecins Sans Frontieres (MSF) provides health services, including TB care to the people who live in the five conflict zones. However, the Ethiopian government denied MSF access to these zones from April 2007 [18]. This study was conducted from June - September 2007, in the Jigjiga and Shinile zones of the SRS of Ethiopia.

## Methods

**Figure 1:**
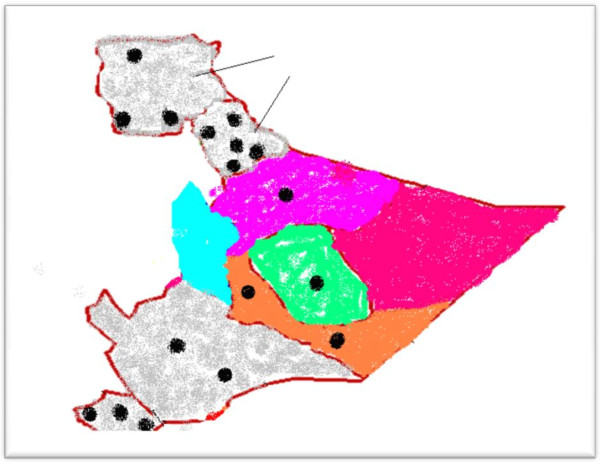
**The distribution of tuberculosis diagnostic centers in the 9 zones of the SRS of Ethiopia**. - Black dots represent diagnostic centers. - Colored areas represent 5 conflict zones, while shadowed areas represent non conflict zones. - Arrow points to the zones studied in this research project.

An institution based, cross sectional study was carried out in the SRS of Ethiopia. The study population was selected from the TB management units in the Jigjiga and Shinile zones of the SRS. The selection of the study sites was based upon security and accessibility, i.e. the existence of roads that could be safely traveled. Moreover, about 50% of the diagnostic facilities in the SRS are found within these two zones (figure 1). As a consequence, pastoral nomads from other zones in the region come to these facilities for the diagnosis and treatment of TB. The respondents were illiterate and unable to read consent forms. As a consequence, witnessed consent was obtained from patients. The study was approved by the Regional Committee for medical Research Ethics.

We calculated the sample size using the formula necessary for determination of sample size required for estimating single proportion. By using previous study on patient delay in the diagnosis of pulmonary TB in Ethiopia which obtained a proportion delay of 82% of more than one month [19] with 95% confidence interval and margin of error of 5%, we found a sample size of 226. Pre-tested structured questionnaires were consecutively administered to 226 patients, all of whom were pastoralists who had been in the intensive phase of TB treatment from June to September 2007. Patients over 15 years of age, who gave their informed consent, were included in the study.

The outcome variable was delay in the diagnosis of TB; referring to a period from the onset of clinical symptoms of TB to the first visit to a professional health care provider. Patients were asked about major symptoms of TB, the length of time that they suffered from these major symptoms and the date when they first visited a professional health care provider. Along with the questionnaire-based interviews, we simultaneously cross-checked the out-patient cards of respondents, their patient registration cards, laboratory registrations and TB registration books. The second outcome variable was 'self-treatment' prior to a biomedical diagnosis. The main predictor variable was area of residence at the onset of major TB symptoms. This was dichotomized as being in 'conflict zones' or in 'non-conflict zones'. Moreover, we collected socio-demographic variables for statistical control, including age, gender, means of livelihood, and the distance to a health facility.

### Analysis

The median of the observed data (60 days) was used as a cut off point for short and long delay in diagnosis of TB [14,19,20]. Frequencies were used to summarize data concerning the different individual characteristics of respondents. Group differences were calculated using Chi-square and Mann-Whitney test. If groups were more than two, we employed Kruskal-Wallis test. These statistical methods were employed because they are appropriate for analysis of categorical variables. Afterwards, logistic regression analysis was performed to assess the associations between outcome variables and the predictor variable. Odd ratios (OR) and 95% confidence intervals (CI) were obtained from each variable. P-value <0.05 was considered statistically significant.

### Results

Two hundred and twenty six TB patients were interviewed from June to September 2007. Among them, 172 (76%) were from non-conflict zones of the region, while 54 patients (24%) were from conflict zones. Socio-demographic characteristics in relation to patients' area of residence are shown in Table 1. The proportion of women and men were 45.1% and 54.9%, respectively. The average age of the study population was 32.2 years (SD ± 13.0 years). The vast majority of the respondents in this study population (200, 88.5%) were illiterate. There was no difference between the two groups with regard to socio-demographics such as gender, age, marital status, occupation and education. However, a higher proportion of patients from conflict zones (63%) were pastoral nomads and the remaining proportion (37%) gained their livelihoods by combining agriculture and pastoralism. The vast majority of study participants believed that TB is caused by malnutrition (78%) and by overworking (79%). Only 27% knew that TB is an infectious disease that is caused by bacteria.

**Table 1:** Demographic characteristics of study population in relation to patients' area of residence (conflict vs non-conflict zones)

Characteristics	From conflict zones		From non-conflict zones	
	No	%	No	%
**Sex**				
Female	24	44.4	78	45.3
Male	30	55.6	94	54.7
**Age**				
≤ 25	17	31.5	70	40.7
26-45	24	44.4	80	46.5
≥ 46	13	24.1	22	12.8
**Education**				
Illiterate	49	90.7	151	87.8
Literate	5	9.3	21	12.2
**Marital status**				
Married	35	64.8	94	54.7
Single	14	25.9	65	37.8
Widowed/Divorced	5	9.3	13	7.6
**Form of TB**				
Pulmonary	36	66.7	139	80.8
Extra-pulmonary	18	33.3	33	19.2

Two hundred and six patients (91%) reported that they consume raw milk, while 89% have never heard that the consumption of raw milk can cause the transmission of TB from cattle to man.

The median delay in diagnosis of 120 days was recorded for patients from conflict zones, which was twice as high as the median delay recorded for patients from non-conflict zones (60 days). This difference was significant (Mann-Whitney test, P < 0.001). For patients from conflict zones, 74% undertook self-treatment prior to diagnosis, while the corresponding proportion of patients undertaking self-treatment in non-conflict zones was 45%. This difference was also significant (Chi-square test, P < 0.001).

The crude logistic regression analysis (table 2) shows that pastoral nomads from conflict zones had significantly greater odds of delay (OR = 4.3; 95% CI: 2.22-8.38) than those from non-conflict zones. This significant difference in delay between the two groups persisted (OR = 3.06; 95% CI: 1.47-6.36) even when adjustments were made for age, gender, livelihood category and distance to health facilities. Similarly, a fully adjusted regression model shows that pastoral nomads from conflict zones had more than 3 times higher odds of undertaking self-treatment prior to diagnosis (OR = 3.34, 95% CI: 1.56-7.12) than their corresponding group.

**Table 2:** Associations between delay in the diagnosis of TB, self-treatment prior to diagnosis, and being an inhabitant of a conflict zone

	Delay in diagnosis		Self treatment	
Variables	Module1 crude	Module 2 adjusted	Module1 Crude	Module2 adjusted
	OR (95% CI)	OR (95% CI)	OR (95% CI)	OR (95% CI)
**Zone of residence**				
Non-conflict Zones	1.00	1.00	1.00	1.00
Conflict Zone	4.32 (2.22-8.38)	3.06 (1.47-6.36)	3.28 (1.66-6.47)	3.34 (1.56-7.12)
**Gender**				
Male		1.00		1.00
Female		1.16(0.65-2.06)		0.61(0.35-1.06)
**Age**				
0-25		1.00		1.00
26-45		0.48(0.26-0.91)		0.63(0.35-1.13)
46+		1.32(0.47-3.69)		0.61(0.23-1.65)
**Pastoralist status**				
Agro-pastoralist		1.00		1.00
Nomadic -pastoralist		2.33(1.28-4.24)		0.81(0.45-1.46)
**Distance to health facility**				
<10 km		1.00		1.00
>10 km		1.30(0.65-2.62)		1.32(0.69-2.53)

Module 1: Only the predictor variable was included in the logistic regression model.

Module 2: Potential confounders were added in the model.

## Discussion

The large numbers of armed conflicts in Africa may impact TB control programs, because armed conflicts interfere with the goals of identifying and curing TB patients [21]. This study reports over 3 times higher odds of delay in the diagnosis of TB for patients from conflict zones of the Somalia Regional State of Ethiopia, when compared to their counterparts from non-conflict zones. Delay in the diagnosis of TB patients has been associated with increased transmission of the disease [22]. Furthermore, conflicts and the resultant restricted access to health services, is known to exacerbate the incidence of tuberculosis [23]. Armed conflicts may not only fuel TB epidemics by escalating poverty and malnutrition, and thereby increase the number of TB susceptible individuals, but such conflicts may also deter infectious TB patients from seeking prompt diagnosis and treatment. This will lengthen the duration of the infectious period and thus increase the pool of infections within communities. Scenarios of this kind were experienced during the war in Iraq, where the number of new TB cases nearly tripled as the war impoverished people, destroyed health infrastructures and interrupted access to anti-TB treatment [24]. A four-fold increase of TB incidence has also been recorded in Bosnia and Herzegovina since the beginning of the war in 1991 [25]. Increased rates of active TB has been associated with conditions of war in a number of reports [26]. In Ethiopia, over a third of the population is exposed to TB and more than 120,000 new cases were reported in the year 2004 alone [27]. Unless action is taken to improve the access to TB diagnosis and treatment, the World Health Organization (WHO) predicts that the number of active TB cases in parts of sub-Saharan Africa will double within 10 years [28]. This is very likely in countries such as Ethiopia, where the vast majority of its people have no access to TB care [29]. As shown in figure 1, there are only 4 diagnostic centers in conflict zones of the Somalia Regional State; an area in which more than 2 million people are inhabitants, obliging TB patients to walk for more than 100 kilometers to access TB care. As health care needs increase in conflict settings, access to health care is often limited by poverty and by the lack of security [30]. This is reflected by extremely long patient delay (median, 120 days) recorded for patients in conflict zones of the Somalia Regional State of Ethiopia. The median delay of 120 days in the diagnosis of TB greatly contrasts with the delay in diagnosis recorded for TB patients from non-conflict zones in the same region (60 days), as well as the recorded delays for patients in other peaceful regions of the country, such as Addis Ababa (60 days) [31], the Amhara region (30 days) [19], the Southern Nations region (30 days) [32] and the Tigre region (30 days) [33]. The finding in this study is consistent with other findings that armed conflicts are a source of diagnostic delay, because they prevent patients from seeking prompt TB care [34].

The 1978 Alma Ata Declaration stated that access to health care for all is a human right, and its violation has been described as being unacceptable, when the causes for that violation are unjust, avoidable and unnecessary [35]. We believe that the armed conflict in the Somalia Regional State of Ethiopia is an avoidable factor that substantially contributes to TB epidemics in Ethiopia. Since there is an imminent danger that conflict zones in Ethiopia may be a breeding ground for TB in that country, international organizations and national authorities should establish programs that are specifically earmarked for the prompt diagnosis and treatment of TB for those people who inhabit areas where armed conflicts continue to be waged.

Our study shows that patients from conflict zones have significantly higher odds of undertaking self treatment (OR = 3.34, 95% CI: 1.56-7.12) compared to patients from non-conflict zones. A previous study shows that 87% of the pastoral nomads who were TB patients in the SRS of Ethiopia sought traditional (animist) health care for their illness prior to diagnosis [14]. For people who have little exposure to modern medical care, an appeal to traditional healers may be the only option. A significant association between self-treatment and long delay in the diagnosis of TB was documented in Ethiopia [19,36]. Moreover, self-treatment was also associated with increased morbidity and death from TB [37]. Armed conflicts hamper TB control efforts not only by disrupting the health system but by diverting economic resources to priorities other than health needs [38]. When national priorities shift and attention is deflected, TB control efforts may suffer [39]. The most cited reason for the increase in TB epidemics in Ethiopia is a lack of sufficient funds for TB control programs [40]. Although underfunding makes the provision of effective intervention difficult [41], sustainable political commitment for the diagnosis and treatment of TB is a fundamental condition for the implementation of successful TB control programs [41]. Being mindful of this, we advocate initiatives that will generate and sustain the effective political will needed to insure that effective TB control will be implemented in the SRS of Ethiopia.

The present study has several potential limitations. Since this was a cross-sectional study, we are unable to determine cause-and-effect relationships. Our outcome variables were necessarily self-reported, and these reports may suffer from recall bias.

## Conclusion

Patients from conflict zones of the SRS of Ethiopia have significantly higher delay in the diagnosis of TB and higher levels of self-treatment prior to diagnosis than patients from non-conflict zones. However, the early diagnosis and treatment of TB patients is possible in conflict settings [42]. This can be achieved by the promotion of peace, by the expansion of user friendly DOTS in conflict zones and by empowering the local community. Local communities need training programs to qualify community health workers for the early detection of TB suspects, and for the distribution and observation of treatment. Moreover, international organizations that provide health services, such as TB care, should be given unconditional access to conflict zones so that people who are affected by the conflict are provided with TB care that is within their reach.

## Competing interests

The authors declare that they have no competing interests.

## Authors' contributions

AG: Did data collection, data analysis and drafted the manuscript. GB: Was involved in data collection, data analysis and in writing the manuscript. All authors read and approved the final version of the manuscript.
